# Performance Improvement of Seismic Response Prediction Using the LSTM-PINN Hybrid Method

**DOI:** 10.3390/biomimetics10080490

**Published:** 2025-07-24

**Authors:** Seunggoo Kim, Donwoo Lee, Seungjae Lee

**Affiliations:** School of Industrial Design & Architectural Engineering, Korea University of Technology & Education, 1600 Chungjeol-ro, Byeongcheon-myeon, Cheonan 31253, Republic of Korea; seunggoo@koreatech.ac.kr (S.K.); leeseung@koreatech.ac.kr (S.L.)

**Keywords:** seismic response prediction, structural dynamics, long short-term memory (LSTM), physics-informed neural network (PINN)

## Abstract

Accurate and rapid prediction of structural responses to seismic loading is critical for ensuring structural safety. Recently, there has been active research focusing on the application of deep learning techniques, including Physics-Informed Neural Networks (PINNs) and Long Short-Term Memory (LSTM) networks, to predict the dynamic behavior of structures. While these methods have shown promise, each comes with distinct limitations. PINNs offer physical consistency but struggle with capturing long-term temporal dependencies in nonlinear systems, while LSTMs excel in learning sequential data but lack physical interpretability. To address these complementary limitations, this study proposes a hybrid LSTM-PINN model, combining the temporal learning ability of LSTMs with the physics-based constraints of PINNs. This hybrid approach allows the model to capture both nonlinear, time-dependent behaviors and maintain physical consistency. The proposed model is evaluated on both single-degree-of-freedom (SDOF) and multi-degree-of-freedom (MDOF) structural systems subjected to the El-Centro ground motion. For validation, the 1940 El-Centro NS earthquake record was used, and the ground acceleration data were normalized and discretized for numerical simulation. The proposed LSTM-PINN is trained under the same conditions as the conventional PINN models (e.g., same optimizer, learning rate, and loss structure), but with fewer training epochs, to evaluate learning efficiency. Prediction accuracy is quantitatively assessed using mean error and mean squared error (MSE) for displacement, velocity, and acceleration, and results are compared with PINN-only models (PINN-1, PINN-2). The results show that LSTM-PINN consistently achieves the most stable and precise predictions across the entire time domain. Notably, it outperforms the baseline PINNs even with fewer training epochs. Specifically, it achieved up to 50% lower MSE with only 10,000 epochs, compared to the PINN’s 50,000 epochs, demonstrating improved generalization through temporal sequence learning. This study empirically validates the potential of physics-guided time-series AI models for dynamic structural response prediction. The proposed approach is expected to contribute to future applications such as real-time response estimation, structural health monitoring, and seismic performance evaluation.

## 1. Introduction

Accurate prediction of a structure’s seismic response—namely displacement, velocity, and acceleration—is crucial for quantitatively assessing the vulnerability of structures under earthquake loading [1]. The simulation of such seismic responses typically relies on solving the equations of motion, which in turn involves the numerical integration of large-scale differential equations using complex algorithms such as time-history analysis [2]. However, traditional approaches often face significant challenges due to high computational costs, strong nonlinearities, and complicated boundary conditions, thereby necessitating the development of alternative methodologies.

With recent advancements in machine learning and artificial intelligence (AI), deep neural networks (DNNs) have emerged as promising tools for solving classical applied mathematics problems such as partial differential equations (PDEs) [3,4]. These data-driven approaches demonstrate potential to overcome the limitations of conventional numerical analysis by learning and predicting the behavior of complex systems. Since the 1990s, artificial neural networks (ANNs), the most fundamental form of DNNs, have been applied to predict structural seismic responses [5,6]. However, as ANNs rely heavily on the quality of training data, their predictive reliability diminishes when data quality is poor. In particular, for nonlinear dynamic systems such as seismic structural responses, acquiring high-quality data is often difficult, which limits the generalization ability of ANN models. Therefore, there is a growing need for alternative prediction methods that can reduce data dependency while improving model reliability.

**Figure 1:**
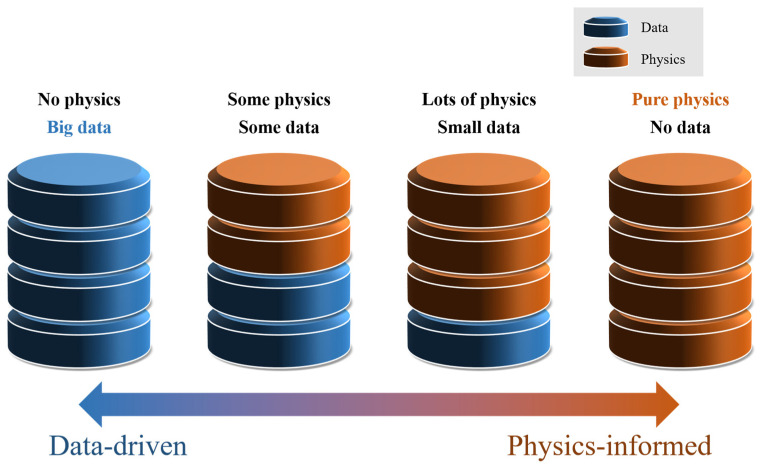
Relationship between the data-driven and physics-informed [6].

In 2019, Raissi et al. proposed the physics-informed neural network (PINN), which integrates physical laws and scientific knowledge into neural networks. This approach allows for effective learning and prediction even with a limited amount of data [7,8,9]. As shown in Figure 1, solution strategies for forward and inverse problems related to PDEs and engineering applications can be categorized into four groups. The right-hand side of the figure corresponds to methods involving PINNs, where numerical computation techniques such as the finite element method (FEM) or finite difference method (FDM) are used to generate synthetic data, and the PINN aims to minimize error based on such data [10,11].

Since its introduction, PINN has seen rapid adoption in various engineering fields. In the architecture and structural domains, ongoing efforts have been made to predict dynamic structural behavior and response using PINN. Guo and Fang (2023) applied PINN to identify parameters of a five-story frame structure and demonstrated that accurate parameter estimation is achievable with limited data [12]. Bolandi et al. (2023) proposed a PINN-based model called PINN-Stress to predict dynamic stress distribution in 2D steel plates in real time, confirming its strong generalization performance [13]. Zhou et al. (2024) applied PINN to predict seismic responses of structures in real time, reporting faster and more accurate predictions compared to conventional finite element analysis [14]. Li et al. (2024) successfully applied PINN to frictional nonsmooth dynamics problems, achieving greater computational efficiency than traditional numerical time integration techniques [15].

As discussed earlier, the use of PINN for reliable structural response prediction and dynamic analysis has been steadily progressing. However, PINN has limitations when it comes to learning time-series information or addressing highly nonlinear dynamic problems in large-scale, complex systems, often resulting in decreased prediction accuracy [16,17]. This shortcoming has led to ongoing demands for the development of improved models, although a fully satisfactory technical solution has yet to be achieved. To address this issue, the present study proposes and validates a hybrid model that combines long short-term memory (LSTM)—a type of recurrent neural network (RNN) capable of learning long-term dependencies—with PINN, in order to enhance the performance of structural dynamic response prediction. LSTM networks are well-suited for time-series data due to their memory-gated architecture, which effectively captures temporal dependencies [18]. Thanks to this capability, LSTM has been successfully integrated with PINN in various domains, yielding promising results [19]. For instance, Cho et al. (2022) developed an LSTM-PINN hybrid model to improve temperature prediction in battery systems and reported enhanced prediction accuracy compared to conventional PINN [20]. Halder et al. (2023) employed LSTM-PINN to predict wave motion even under conditions of extremely limited training data [21]. Dou et al. (2025) proposed an LSTM-PINN model to predict flow fields around a two-dimensional circular cylinder, and demonstrated improvements in both prediction accuracy and computational efficiency over traditional PINN models [22]. While the advantages of LSTM-PINN have been demonstrated in various domains, its application to complex structural systems—particularly seismic response prediction involving high-dimensional and time-dependent behaviors remains limited. In this context, recent LSTM-based studies have addressed structural dynamics: Tian et al. (2021) applied BiLSTM to estimate cable tension; Liao et al. (2023) used attention-based LSTM for seismic response modeling [23,24]. These cases highlight the growing potential of advanced time-series models in addressing the challenges of structural response prediction under complex conditions. However, seismic loads are inherently nonlinear and irregular in both space and time, making it difficult for standalone PINNs to achieve generalized predictions. Therefore, this study introduces the LSTM-PINN hybrid model for seismic response prediction, aiming to improve time-series prediction accuracy while simultaneously maintaining physics-informed reliability.

The remainder of this paper is organized as follows. Section 2 describes the framework of the proposed LSTM-PINN model for seismic response prediction. Section 3 explains the numerical methods and example structure used in this study. Section 4 presents and compares the dynamic response results obtained using both PINN and LSTM-PINN. Finally, Section 5 concludes the study.

## 2. LSTM-PINN Framework

In this study, we propose a hybrid architecture called LSTM-PINN, which combines long short-term memory (LSTM)—known for its strength in learning long-term dependencies in time-series data—with the physics-informed neural network (PINN), which incorporates physical laws as constraints directly into the training process. This approach is designed to address two critical limitations: deep learning models often fail to ensure physical consistency in predictions, while physics-based models typically struggle to capture the sequential characteristics inherent in time-series data.

### 2.1. LSTM Network

The most basic form of neural network, the artificial neural network (ANN), is inspired by the structure and behavior of neurons in the human brain [25]. An ANN consists of an input layer, one or more hidden layers, and an output layer. Each hidden layer is composed of neurons. When two hidden layers are used, the output of the first hidden layer is calculated as in Equation (1), and the resulting hidden representation h is then passed to the output layer, where the final predicted value ($\hat{y}$) is obtained as shown in Equation (2). Here, X denotes the input vector, W is the weights, b is the bias terms, $\sigma \left(\cdot \right)$ is the activation function, and $f\left(\cdot \right)$ is the activation function used in the output layer.

(1) $$h=\sigma \left(W_{1}X+b_{1}\right)$$

(2) $$\hat{y}=f\left(W_{2}h+b_{2}\right)$$

**Figure 2:**
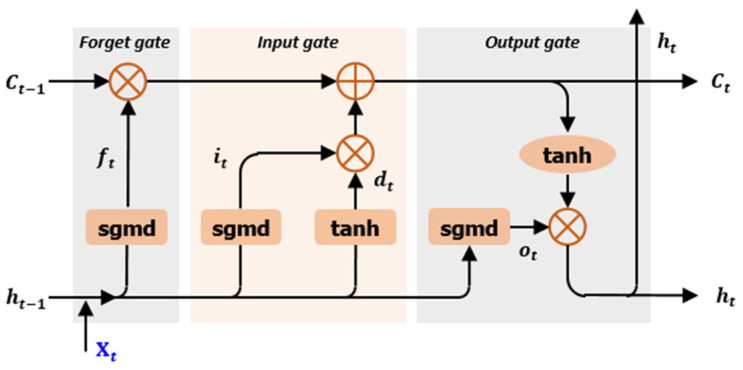
Structure of LSTM network.

Recurrent neural networks (RNNs) are designed to process sequential data, making them well-suited for modeling time-series data such as structural component histories and response records in earthquake engineering. In particular, long short-term memory (LSTM) networks are capable of effectively learning complex temporal patterns and addressing the long-term dependency problem inherent in standard RNNs. The structure of an LSTM network is illustrated in Figure 2 [26,27]. The key innovation of LSTM lies in its two structural components: gates and a memory cell, which enable the network to manage long-term dependencies efficiently. Gates regulate the flow of information, while the memory cell stores or updates information over time, allowing for long-term memory retention. There are three primary types of gates—forget gate, input gate, and output gate—which interact to control and preserve the cell state [28,29]. The function of each gate is described below [30]:

- Forget Gate: Determines which parts of the previous cell state $C_{t-1}$ should be discarded or retained. It takes the previous hidden state $h_{t-1}$ and the current input $X_{t}$ to compute a forget vector $f_{t}$, where each value ranges between 0 (complete forgetting) and 1 (complete retention). The vector $f_{t}$ is then element-wise multiplied with $C_{t-1}$ to selectively erase irrelevant information from the memory cell.
- Input Gate: Selects which incoming information will be stored in the cell state. A sigmoid function generates the input gate signal $i_{t}$, and a hyperbolic tangent function creates a candidate vector $d_{t}$. These are combined to update the cell state, determining which new information to remember.
- Output Gate: Determines what information from the cell state will be output. It uses a sigmoid function to compute the output gate signal $o_{t}$, multiplies it by the tanh-transformed cell state, and produces the final hidden output $h_{t}$. This defines what information is exposed at each time step.

The computations for each of these gate operations at time step t, given input $X_{t}$, are defined as follows in Equations (3)–(8).

(3) $$f_{t}=sgmd\left(W_{f}\cdot \left[h_{t-1},X_{t}\right]+b_{f}\right)$$

(4) $$i_{t}=sgmd\left(W_{i}\cdot \left[h_{t-1},X_{t}\right]+b_{i}\right)$$

(5) $$d_{t}=tanh\left(W_{d}\cdot \left[h_{t-1},X_{t}\right]+b_{d}\right)$$

(6) $$C_{t}=f_{t}⊙C_{t-1}+i_{t}⊙d_{t}$$

(7) $$O_{t}=sgmd\left(W_{O}\cdot \left[h_{t-1},X_{t}\right]+b_{O}\right)$$

(8) $$h_{t}=O_{t}⊙tanh\left(C_{t}\right)$$

LSTM utilizes two primary activation functions that serve complementary roles. The sigmoid function acts as a gate controller in the forget, input, and output gates, selectively regulating the flow of information by determining what to retain, update, or output. In contrast, the tanh function is used for generating candidate states and normalizing the cell state, which helps maintain data balance and alleviates the vanishing gradient problem. Through the combination of these activation functions, LSTM can learn long-term dependencies effectively while managing information flow in a stable and controlled manner. This sophisticated mechanism enables LSTM to capture and predict patterns in time-series data with long-range temporal dependencies.

### 2.2. LSTM-PINN

Physics-Informed Neural Networks (PINNs) are fundamentally designed to incorporate governing physical equations explicitly into the neural network training process. Unlike conventional data-driven approaches, PINNs embed physical constraints directly into the loss function, thereby ensuring that the predicted results remain physically consistent and compliant with the underlying laws of physics.

In this study, we focus on learning the dynamic responses—displacement, velocity, and acceleration—of a single-degree-of-freedom (SDOF) structural system. The governing equation residuals are embedded into the loss function so that the neural network can learn the underlying physical laws directly. PINNs leverage physics-based loss functions to maintain generalization capability even in data-scarce scenarios, and they demonstrate stable predictive performance even when the data contains a certain level of noise [7,31,32]. This is particularly effective for dynamic systems, where physical behavior is well-defined and can be directly incorporated into the loss function to enhance prediction accuracy. As a result, the PINN model can generate physically plausible predictions and accurately capture the behavior of dynamic systems [33].

The total loss function $L_{Total}$ of the PINN model is defined as follows in Equation (9), consisting of a physics-based loss term $L_{G}$ and an initial condition loss term $L_{IC}$. This composition allows the model to reflect both the governing physical principles and the system’s initial conditions. The physics-based loss $L_{G}$ includes the residuals of three physical quantities—displacement, velocity, and acceleration—as shown in Equation (10), enabling the model to capture the interrelationships among these quantities more accurately.

- $L_{displacement}$: Represents the basic motion state of the system and contributes to learning positional information.
- $L_{velocity}$: Reflects the time derivative of displacement and indicates the current motion of the system.
- $L_{acceleration}$: Represents the time derivative of velocity, which reflects the effects of external forces acting on the system.

Each component of the loss function reflects the unique characteristics and interdependencies of the physical quantities involved. Their combination enables the network to learn the dynamic behavior of the system with higher accuracy.

(9) $$L_{Total}=L_{G}+L_{IC}$$

(10) $$L_{G}=L_{displacement}+L_{velocity}+L_{acceleration}$$

**Figure 3:**
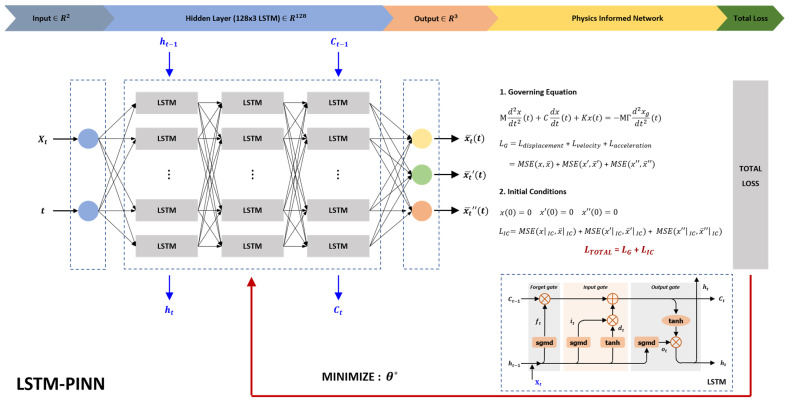
Structure of LSTM-PINN.

Through the loss function configuration described above, the LSTM-PINN model can simultaneously capture the temporal characteristics of time-series data and enforce compliance with physical laws. In particular, for the prediction of dynamic system behavior, considering both temporal dependencies and physical constraints allows the model to achieve high accuracy and physical consistency [34]. This structure is illustrated in Figure 3, which visually explains the components and information flow of LSTM-PINN.

## 3. Numerical Model

**Figure 4:**
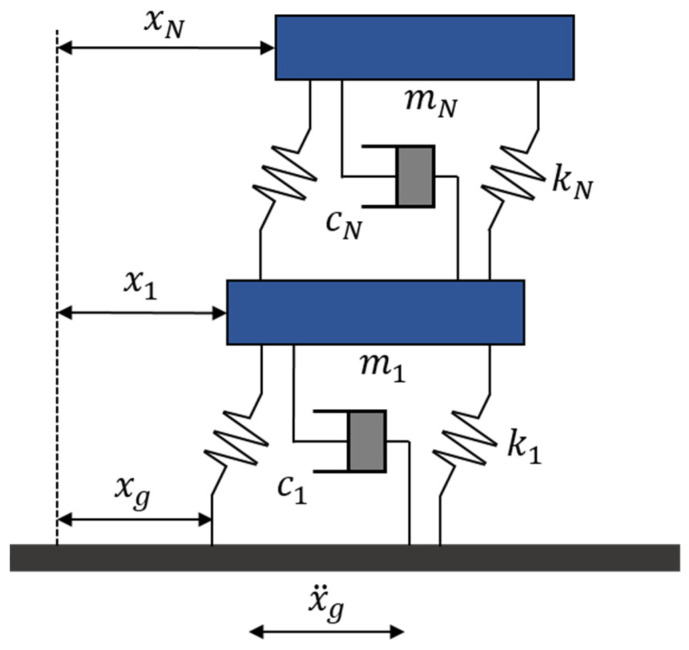
Model of MDOF structure system.

This study adopted two structural analysis models based on the framework proposed by Sadek et al. (1997) [35]. The first is a single-degree-of-freedom (SDOF) system and the second is a 10-story multi-degree-of-freedom (MDOF) system. The structural configuration used in this study is shown in Figure 4. The equations of motion for an N-story MDOF system subjected to seismic loads are expressed in Equation (11):

(11) $$M\ddot{u}\left(t\right)+C\dot{u}\left(t\right)+Ku\left(t\right)=-M{\left\{1\right\}}_{N\times 1}\ddot{x_{g}}\left(t\right)$$

Here, M, C, and K denote the mass, damping, and stiffness matrices of the structure, respectively. The vectors u(t), $\dot{u}(t)$, and $\ddot{u}(t)$ represent the displacement, velocity, and acceleration responses of the structure. The term $\ddot{x_{g}}\left(t\right)$ refers to the ground acceleration input. The matrices M, C, and K are each of size N×N, while the response vectors u(t), $\dot{u}(t)$, and $\ddot{u}(t)$ are of size N×1.

The equation of motion described above is transformed into a state-space representation, and numerical analysis is performed using the fourth-order Runge–Kutta (RK4) method. RK4 is an enhanced version of the Euler method and can be defined by Equations (12)–(16). It estimates the system’s next state by computing four intermediate slopes at each time step and taking their weighted average. This method does not require the explicit computation of higher-order derivatives and can accurately approximate the solution up to the fourth-order term of a Taylor series expansion.

By evaluating four slopes and averaging their influence, RK4 offers significantly improved accuracy over the Euler method. Additionally, it exhibits excellent numerical stability, allowing for the use of larger time steps (∆t), which contributes to improved computational efficiency.

(12) $$k_{1}=f\left(t_{n},y_{n}\right)$$

(13) $$k_{2}=f\left(t_{n}+\frac{∆t}{2},y_{n}+\frac{∆t}{2}k_{1}\right)$$

(14) $$k_{3}=f\left(t_{n}+\frac{∆t}{2},y_{n}+\frac{∆t}{2}k_{2}\right)$$

(15) $$k_{4}=f\left(t_{n}+∆t,y_{n}+∆t\cdot k_{2}\right)$$

(16) $$y_{n+1}=y_{n}+\frac{∆t}{6}\left(k_{1}+2k_{2}+2k_{3}+k_{4}\right)$$

To efficiently solve the second-order differential equation presented in Equation (12), we introduce an auxiliary variable as defined in Equation (17), thereby transforming the system into a set of first-order coupled differential equations, as shown in Equation (18).

(17) $$z=\left[\begin{array}{c} u(t)\\ \dot{u}(t) \end{array}\right]$$

(18) $$\frac{dz}{dt}\left(t\right)=f\left(z,t\right)$$

At this point, the function f(z,t) in the state-space formulation corresponds to the structural acceleration derived from the original equation of motion shown in Equation (11). For example, in the case of a single-degree-of-freedom (SDOF) system, this function can be expressed as:

(19) $$f\left(z,t\right)=\frac{1}{M}\left[-C\;\dot{u}\left(t\right)-K\;u\left(t\right)-M\;\ddot{x_{g}}(t)\right],\;\mathrm{where}\;z=\left[\begin{array}{c} u(t)\\ \dot{u}(t) \end{array}\right]$$

This definition enables the RK4 method to evaluate acceleration at each time step based on displacement and velocity. The same formulation can be extended to MDOF systems using matrix notation.

**Figure 5:**
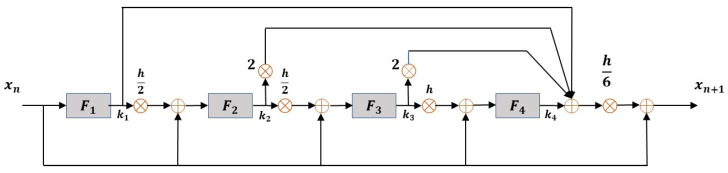
RK4 flowchart.

Using the RK4 method, the primary structural response variables—such as displacement and velocity—can be numerically evaluated by applying the iterative formulas defined in Equations (20) and (21). Here, ∆t denotes the time step size. The details of the computations in Equations (20) and (21) are clarified using Table 1, which presents the intermediate values at each time increment. This numerical analysis framework is illustrated in Figure 5, which demonstrates how the dynamic response of a structure subjected to seismic loading can be stably and accurately predicted.

(20) $$x_{i+1}=x_{i}+\frac{∆t}{6}\left(Z_{1}+2Z_{2}+2Z_{3}+Z_{4}\right)$$

(21) $$Z_{i+1}=Z_{i}+\frac{∆t}{6}\left(F_{1}+2F_{2}+2F_{3}+F_{4}\right)$$

## 4. Results and Discussion

To evaluate the performance of the proposed LSTM-PINN model, we conducted a comparative analysis against the conventional PINN model under the same training conditions of 10,000 epochs. Additionally, to validate the robustness of the proposed method, another comparison was made using a PINN model trained for 50,000 epochs. The PINN models trained for 10,000 and 50,000 epochs are referred to as PINN-1 and PINN-2, respectively. The key training parameters used for each model are summarized in Table 2. To ensure a fair comparison among the three models (PINN-1, PINN-2, and LSTM-PINN), consistent initial training conditions were applied across all models. Specifically, we used the same learning rate (0.001), optimizer (Adam), activation function (tanh), and loss function structure that includes displacement, velocity, and acceleration terms. Although the number of training epochs varied across models, this variation was intentional and tailored to reflect the intrinsic characteristics and convergence behaviors of each model. In particular, the PINN-2 model was trained for a greater number of epochs as an experimental adjustment to compensate for the conventional PINN’s limited ability to capture time-dependent patterns. This measure ensured stable convergence and enabled a fair comparison among all models. To further avoid overfitting and entrapment in local minima, all models incorporated an exponential decay learning rate schedule and an early stopping condition based on the stagnation of validation loss. These strategies contributed to stable convergence and reliable performance across models.

Moreover, the PINN architecture is inherently designed to minimize the risk of converging to physically invalid local minima by incorporating governing differential equations and initial conditions directly into the loss function—as detailed in Equations (9) and (10) and illustrated in Figure 3. This physics-informed structure steers the learning process toward physically meaningful solutions beyond mere data fitting. Consequently, the integration of consistent training conditions and physics-based constraints contributed to both fair model evaluation and robust convergence. Model performance was assessed based on the results obtained using the RK4 method, which served as the reference solution. The evaluation metrics included the mean error and mean squared error (MSE) of the maximum responses (displacement, velocity, and acceleration) at each floor level. The mean error and MSE were computed using Equations (22) and (23), respectively. In these equations, x represents the RK4 reference value, $\bar{x}$ denotes the model prediction, and $n_{d}$ is the total number of data points.

(22) $$Mean\;error=\frac{1}{n_{d}}\sum_{i=1}^{n_{d}}\left|x-{\bar{x}}_{i}\right|$$

(23) $$MSE=\frac{1}{n_{d}}\sum_{i=1}^{n_{d}}{\left(x-{\bar{x}}_{i}\right)}^{2}$$

**Figure 6:**
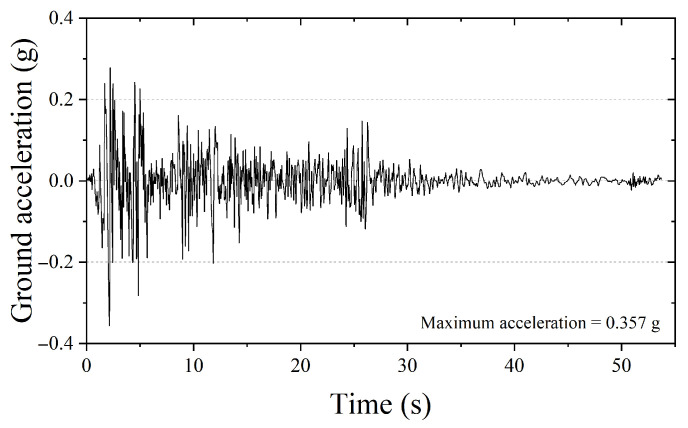
Seismic record of El-Centro NS (1940).

In addition, the predictive accuracy of each model over the entire time history was compared to analyze their respective advantages and limitations. The seismic input used for both the SDOF and MDOF structural models was the El-Centro North–South (NS) ground motion recorded in 1940, as shown in Figure 6. The El-Centro NS record has a total duration of 53.72 s and a peak ground acceleration (PGA) of 0.357 g.

### 4.1. SDOF Structure

**Figure 7:**
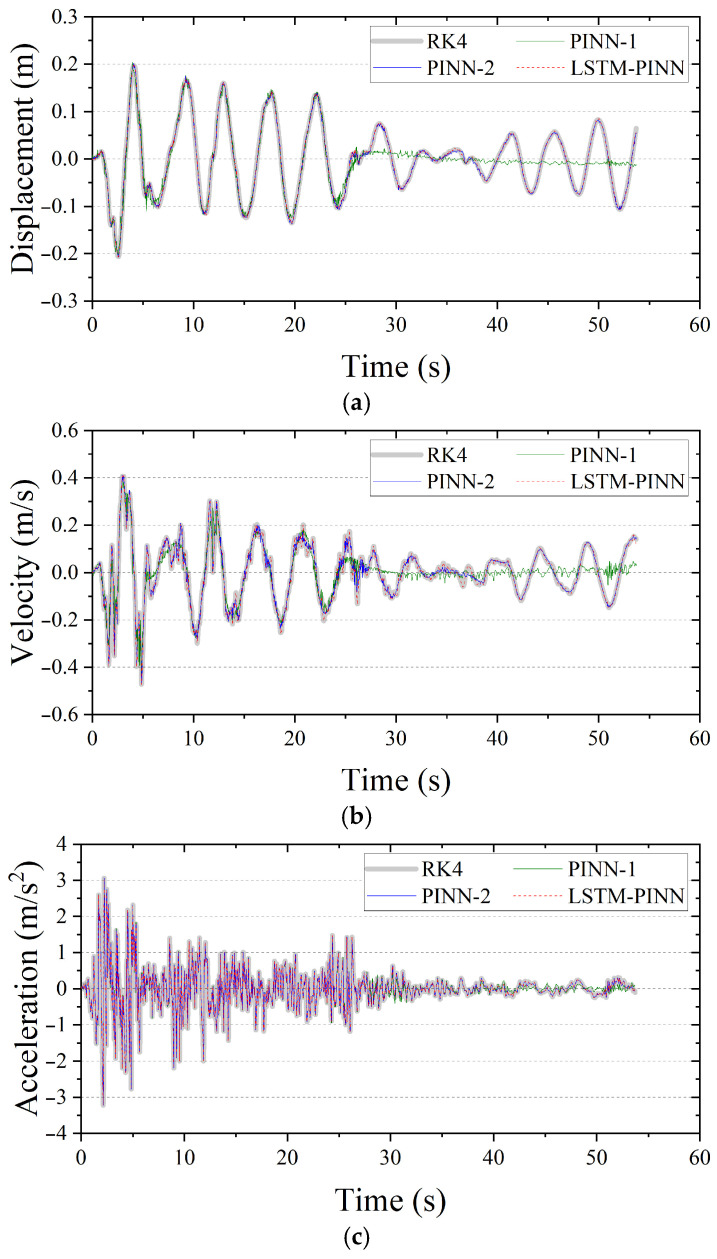
Seismic response of the SDOF structure. (**a**) Displacement; (**b**) velocity; (**c**) acceleration.

For the SDOF structure, the mass M, damping coefficient C, and stiffness K were set to 1.0 ton, 2.0 kN/m, and 0.04 kN·s/m, respectively. Figure 7 presents the displacement, velocity, and acceleration responses predicted by RK4, PINN, and LSTM-PINN for the SDOF system. In Figure 7a, which shows the displacement response, PINN-1 produces results that closely match the RK4 solution up to approximately 25 s, but a noticeable phase shift emerges beyond that point. PINN-2, trained with a greater number of epochs, generally follows the RK4 solution well, although a minor phase difference is observed in the 30–40 s interval. In contrast, the LSTM-PINN demonstrates a high level of agreement with the RK4 results over the entire time duration, showing consistent accuracy in both phase and amplitude prediction. Figure 7b presents the velocity response. Again, PINN-1 performs reasonably well up to around 25 s, but its predictive accuracy rapidly deteriorates afterward. While PINN-2 yields velocity predictions that generally align with the RK4 results, discrepancies appear in intervals with rapidly changing velocities, particularly between 10 and 40 s. In comparison, the LSTM-PINN maintains strong consistency with the RK4 solution throughout the entire time domain. Finally, Figure 7c shows the acceleration response. All models—PINN-1, PINN-2, and LSTM-PINN—exhibit overall agreement with the RK4 results. This may be attributed to the fact that acceleration is explicitly defined as a linear combination of displacement and velocity, making it inherently more predictable. Additionally, since the acceleration term is directly included in the loss function, the models tend to converge toward accurate predictions early in training. However, PINN-1 shows a gradual divergence from the RK4 solution in the latter portion of the time domain.

**Figure 8:**
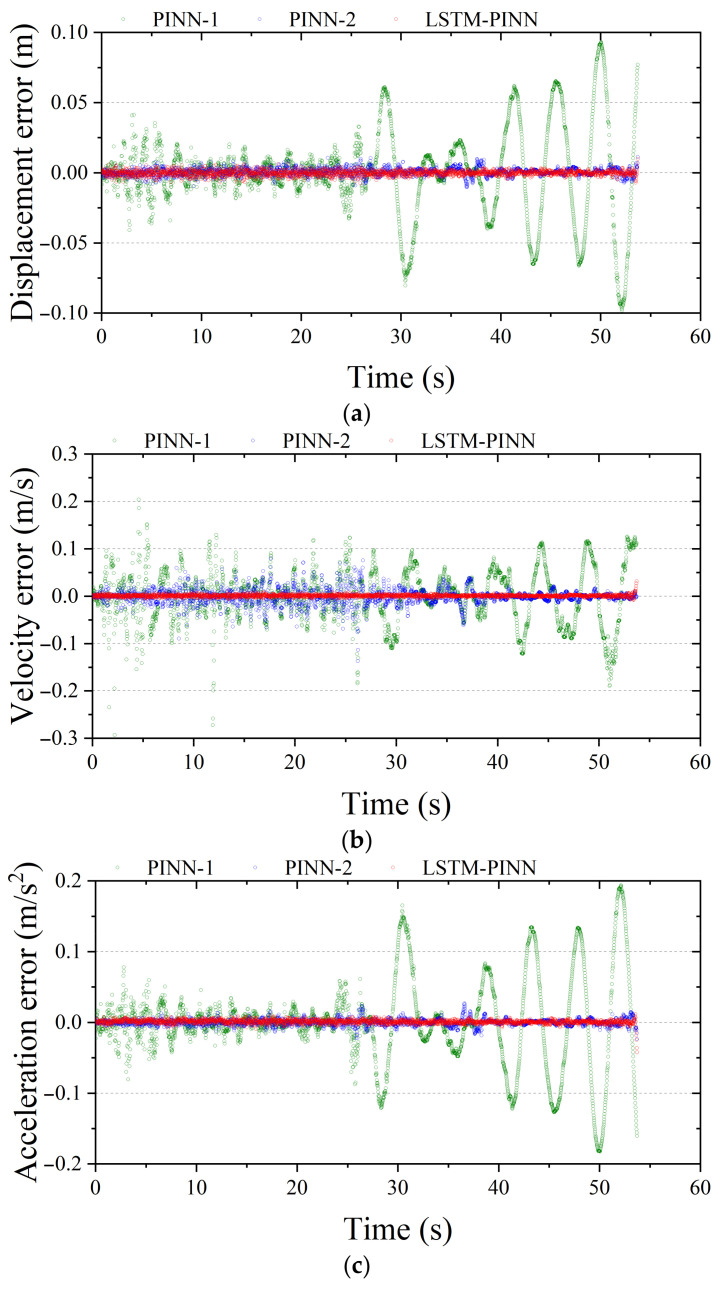
Fitting error analysis of the SDOF structure. (**a**) Displacement; (**b**) velocity; (**c**) acceleration.

Figure 8 visualizes the time-series residuals between the predicted responses of each model and the RK4 reference solution. A residual value closer to zero indicates higher prediction accuracy. Among the three models, PINN-1 exhibits the largest overall error distribution across all physical quantities—displacement, velocity, and acceleration. In particular, after 25 s, significant phase shifts and accumulated amplitude deviations are observed. These discrepancies can be attributed to the accumulation of nonlinear prediction errors during long-term simulation and to limitations in the network’s ability to capture temporal dependencies. PINN-2, which was trained for more epochs, shows a generally reduced error magnitude compared to PINN-1 and maintains relatively stable performance. However, as seen in Figure 8b, temporary error spikes occur in periods of rapid ground motion variation—especially between 10 and 40 s—suggesting sensitivity to transient input fluctuations. In contrast, the LSTM-PINN demonstrates the narrowest and most uniform error distribution among the three models. It maintains a high level of agreement with the RK4 solution throughout the entire time domain without significant error accumulation. Notably, LSTM-PINN achieves this high accuracy with fewer training epochs, which can be attributed to the recurrent neural architecture’s ability to effectively learn temporal dependencies. Therefore, the LSTM-PINN model not only delivers high predictive accuracy but also exhibits superior training efficiency.

Table 3 presents a quantitative comparison of prediction accuracy for the SDOF structure across all models, using mean error and mean squared error (MSE) as evaluation metrics. The mean error represents the average difference between each model’s prediction and the RK4 reference at every time step, while the MSE is the average of the squared differences, providing a more sensitive measure of large deviations. In displacement prediction, LSTM-PINN achieved the lowest mean error of 3.40 × 10^−6^, and both PINN-2 and LSTM-PINN recorded an MSE of 0.00 × 10^−0^. For velocity prediction, LSTM-PINN also exhibited the lowest errors among the three models, with a mean error of 5.97 × 10^−6^ and an MSE of 1.00 × 10^−9^. These results suggest that the LSTM-PINN effectively learns the temporal dependencies inherent in time-series data, contributing to improved predictive stability and convergence behavior. In contrast, PINN-1 exhibited the highest error values across all metrics. Although PINN-2 showed improved performance due to increased training epochs, it still demonstrated a notable performance gap compared to LSTM-PINN in certain evaluation metrics.

### 4.2. MDOF Structure

The corresponding M, C, and K matrices for the MDOF structure are listed in Table 4. In multi-degree-of-freedom (MDOF) systems, the responses of the bottom story and the top story are of particular importance. The bottom story is directly connected to the ground and is the first point at which seismic waves are transmitted into the structure, thereby influencing the overall vibration pattern. On the other hand, the top story generally experiences the largest displacement and plays a critical role in determining the structure’s overall dynamic behavior and safety. Accordingly, this study focuses on analyzing the displacement, velocity, and acceleration responses of the bottom and top stories. Response results for intermediate floors are provided in Appendix A and Appendix B.

**Figure 9:**
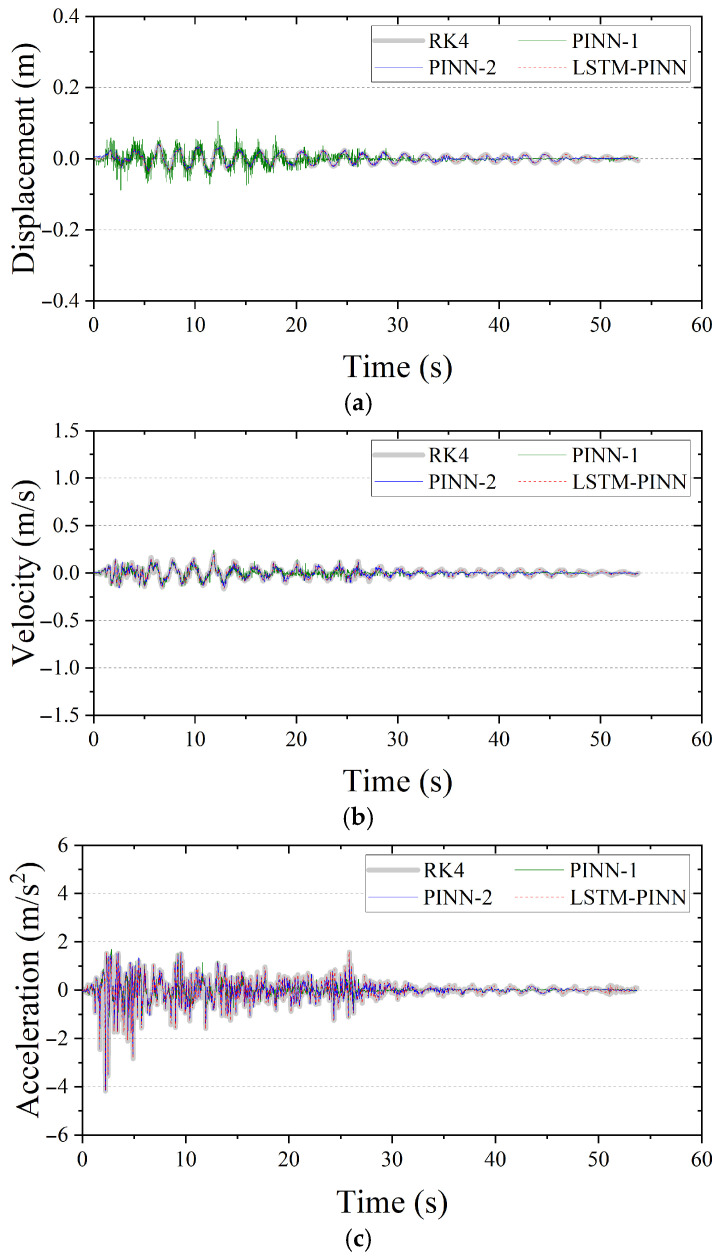
Seismic response of the first floor in MDOF structure. (**a**) Displacement; (**b**) velocity; (**c**) acceleration.

**Figure 10:**
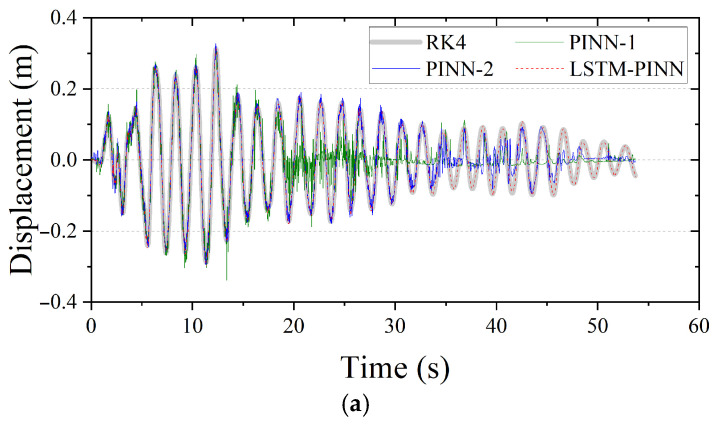

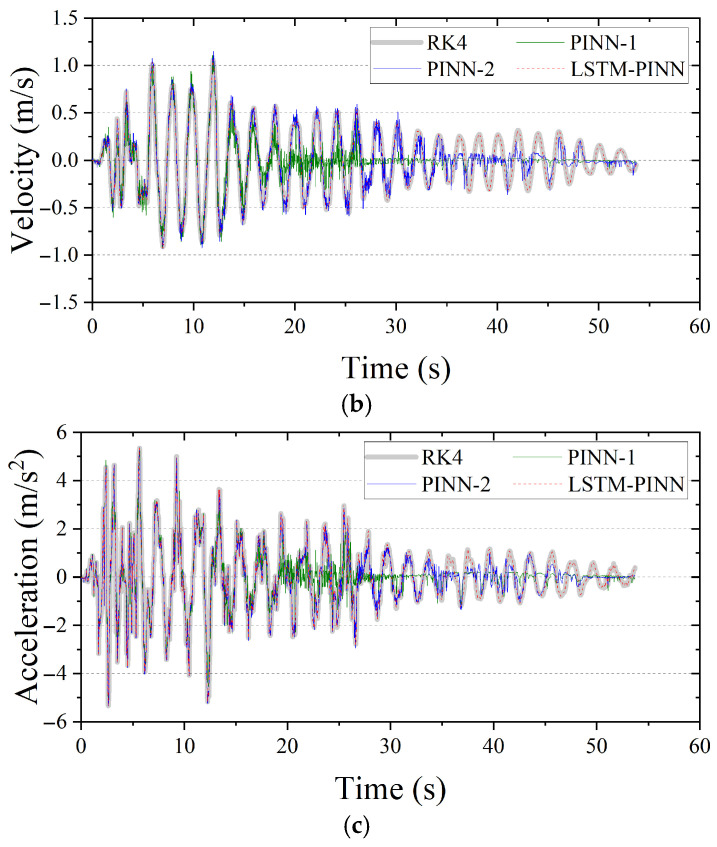
Seismic response of the top floor in MDOF structure. (**a**) Displacement; (**b**) velocity; (**c**) acceleration.

Figure 9 and Figure 10 present the displacement, velocity, and acceleration responses of the first and top stories of the MDOF system, respectively, as predicted by the RK4 method, PINN, and LSTM-PINN. As in the SDOF case, PINN-1 and LSTM-PINN were trained for 10,000 epochs, while PINN-2 was trained for 50,000 epochs. According to the results in Figure 9, the LSTM-PINN shows the highest agreement with the RK4 solution across all response variables—displacement, velocity, and acceleration—and demonstrates excellent phase and amplitude consistency. In contrast, both PINN-1 and PINN-2 exhibit noticeable phase differences relative to the RK4 results. Particularly during the early vibration phase, their predicted responses show significant instability and noise. In the top story responses shown in Figure 10, LSTM-PINN accurately captures the damping characteristics and variations in the vibration period. It provides the most precise tracking of inflection points and oscillation cycles across all physical quantities. By comparison, PINN-1 suffers a sharp decline in prediction accuracy during the initial phase, resulting in exaggerated and irregular oscillatory patterns. PINN-2 also shows distortion in both phase and amplitude responses beyond approximately 35 s. These findings highlight the limitations of conventional PINN models in handling complex, temporally-dependent MDOF systems. At the same time, they clearly demonstrate that the LSTM-PINN, through its ability to capture temporal dependencies effectively, significantly improves both the accuracy and stability of seismic response predictions.

**Figure 11:**
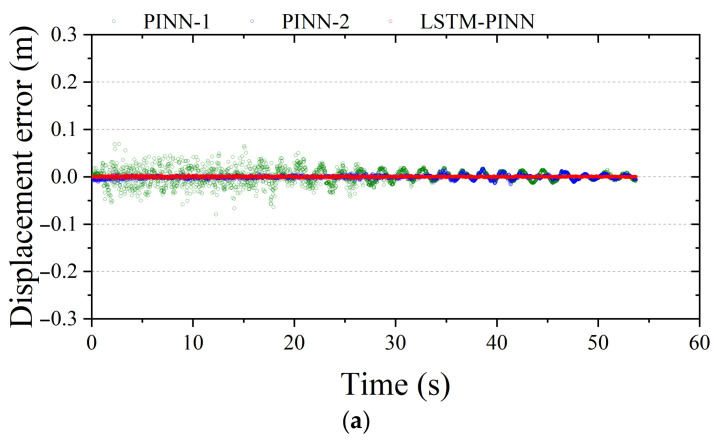

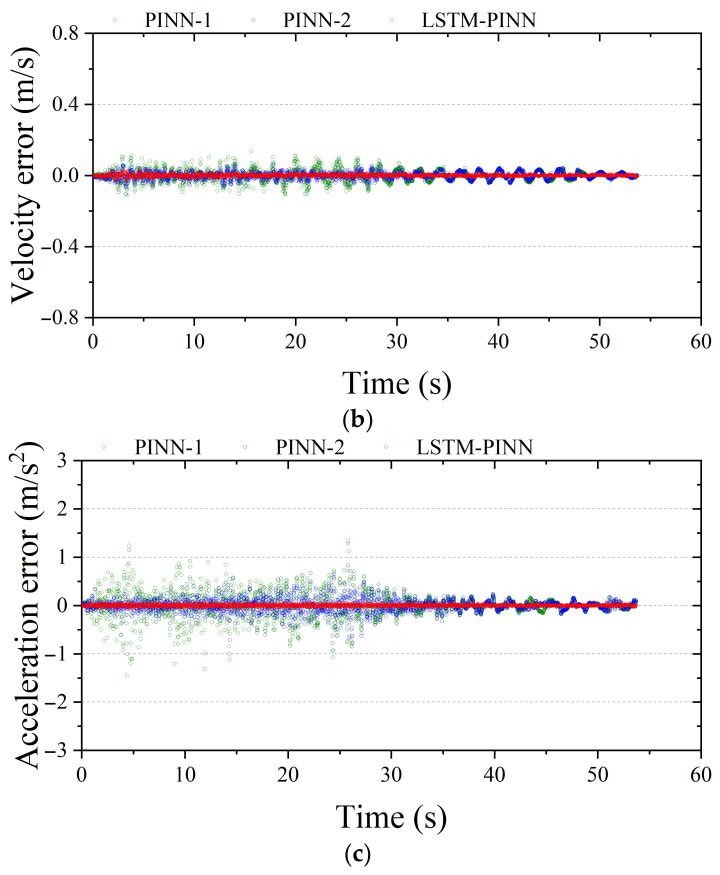
Fitting error analysis of the first floor in MDOF structure. (**a**) Displacement; (**b**) velocity; (**c**) acceleration.

**Figure 12:**
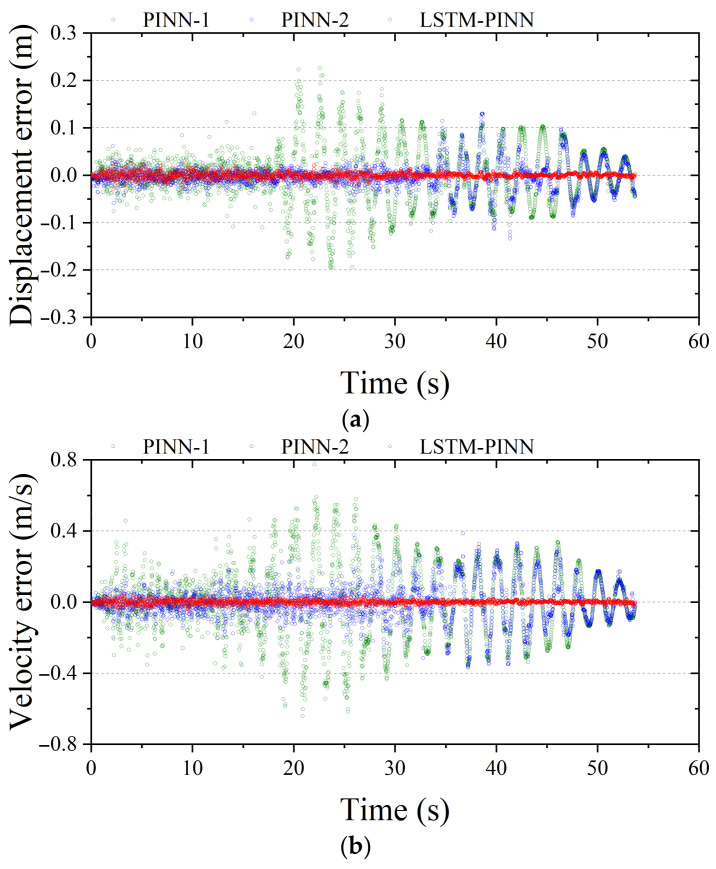

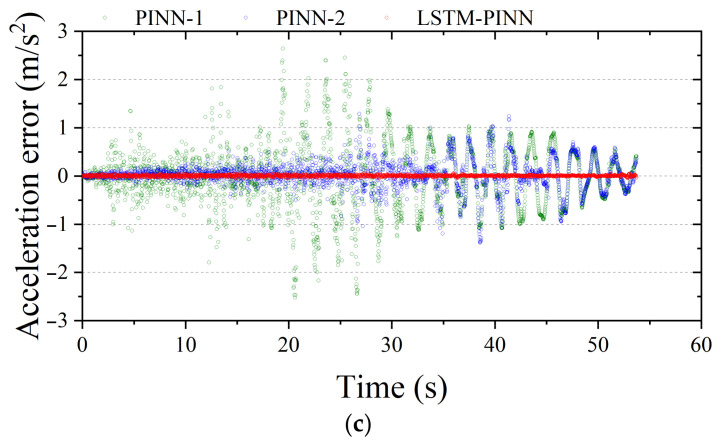
Fitting error analysis of the 10th floor in MDOF structure. (**a**) Displacement; (**b**) velocity; (**c**) acceleration.

Figure 11 and Figure 12 show the response errors for the first and tenth stories of the MDOF system, respectively. In all results presented in Figure 11, PINN-1 exhibits the largest error among the models. Notably, it shows relatively large errors during the initial vibration phase, which gradually stabilize over time. PINN-2, while showing reduced error compared to PINN-1, still demonstrates prominent errors in the early vibration period. In contrast, LSTM-PINN maintains the smallest error throughout the entire time domain, indicating the most stable performance among the three models. In Figure 12, the response becomes more complex due to higher structural dynamics, and the prediction error tends to increase accordingly, depending on the model’s capability. Both PINN-1 and PINN-2 show a trend of increasing error in the later stages of the vibration period. Meanwhile, LSTM-PINN consistently maintains the lowest error across the entire duration and delivers stable results across all response variables. These findings indicate that the LSTM-PINN achieves high reliability even in predicting higher-order responses, making it more suitable for complex structural systems with significant dynamic behaviors.

Table 5 and Table 6 present the mean error and mean squared error (MSE) results for the first and tenth stories of the MDOF structure, respectively, as predicted by each model. For displacement prediction, LSTM-PINN achieved the lowest mean errors of 8.04 × 10^−7^ and 3.17 × 10^−5^ (1st and 10th stories). Likewise, it recorded the lowest MSE values: 3.57 × 10^−12^ and 5.63 × 10^−9^, respectively. In velocity prediction, LSTM-PINN again outperformed the other models, with mean errors of 2.89 × 10^−5^ and 1.13 × 10^−4^, and MSE values of 5.53 × 10^−9^ and 5.55 × 10^−8^, for the first and tenth stories, respectively. For acceleration prediction, LSTM-PINN yielded the lowest mean errors of 2.22 × 10^−4^ and 2.66 × 10^−4^, along with the lowest MSE values of 1.90 × 10^−7^ and 2.44 × 10^−7^, for the respective floors. These results, consistent with the findings from the SDOF case, highlight the LSTM-PINN’s ability to effectively learn the temporal dependencies in time-series data. Furthermore, compared to the SDOF system, all models—particularly LSTM-PINN—demonstrated improved accuracy in both mean error and MSE under the more complex MDOF conditions. This confirms that as the complexity of the structural dynamics increases, LSTM-PINN consistently outperforms conventional PINNs in both accuracy and robustness.

## 5. Conclusions

In this study, a novel LSTM-PINN model was proposed to predict the dynamic structural responses—displacement, velocity, and acceleration—under seismic loading. While conventional PINN models are advantageous in reducing data dependency by incorporating physical laws into the learning process, they have limitations in capturing sequential dependencies inherent in time-series data. To address this issue, the LSTM architecture, which is well-suited for modeling temporal patterns, was integrated into the PINN framework to enhance predictive performance for complex structural responses.

The proposed LSTM-PINN model was evaluated using both single-degree-of-freedom (SDOF) and multi-degree-of-freedom (MDOF) structures, with the numerical solutions obtained via the RK4 method serving as reference data. Its performance was compared with two baseline PINN models (PINN-1 and PINN-2) using various error metrics. The results showed that LSTM-PINN consistently achieved the lowest mean error and mean squared error across all response variables. In particular, it exhibited high predictive stability in the latter part of the simulation and in higher-order derivative predictions. Furthermore, the model maintained robust accuracy even in capturing complex nonlinear responses at the top stories, demonstrating enhanced generalization capability.

The findings of this study empirically demonstrate the advantages of the LSTM-PINN model in physics-informed prediction tasks that involve strong temporal dependencies. The proposed framework shows promising potential for applications such as real-time structural response forecasting, structural health monitoring, and seismic-resistant design. Future research will include ablation studies that compare the performance of PINN-only, LSTM-only, and LSTM-PINN models under identical conditions, in order to more clearly validate the effectiveness of the hybrid approach. In addition, nonlinear material behaviors, uncertainty quantification methods, and various real earthquake records will be incorporated in future work to further improve the practicality and generalizability of the proposed model.

## Figures and Tables

**Table 1:** State variable computations at each RK4 substep ($x,\dot{x},\ddot{x}$).

Time (t)	Displacement (x)	$\mathrm{Velocity}\;(z=\frac{dx}{dt})$	$\mathrm{Acceleration}\;(F=\frac{dz}{dt}=\frac{d^{2}x}{dt^{2}})$
$T_{1}=t_{i}$	$X_{1}=x_{i}$	$Z_{1}=z_{i}$	$F_{1}=F\left(X_{1},Z_{1},T_{1}\right)$
$T_{2}=t_{i}+\frac{∆t}{2}$	$X_{2}=x_{i}+\frac{∆t}{2}\cdot Z_{1}$	$Z_{2}=z_{i}+\frac{∆t}{2}\cdot F_{1}$	$F_{2}=F\left(X_{2},Z_{2},T_{2}\right)$
$T_{3}=t_{i}+\frac{∆t}{2}$	$X_{3}=x_{i}+\frac{∆t}{2}\cdot Z_{2}$	$Z_{3}=z_{i}+\frac{∆t}{2}\cdot F_{2}$	$F_{3}=F\left(X_{3},Z_{3},T_{3}\right)$
$T_{4}=t_{i}+∆t$	$X_{4}=x_{i}+∆t\cdot Z_{3}$	$Z_{4}=z_{i}+∆t\cdot F_{3}$	$F_{4}=F\left(X_{4},Z_{4},T_{4}\right)$

**Table 2:** Parameters of each analysis model.

Model	Parameters
PINN-1	Epochs: 10,000; Hidden layers: 3; Neurons/layer: 128; Activation function: tanh; Learning rate: 0.001; Optimizer: Adam; Loss: displacement, velocity, acceleration
PINN-2	Epochs: 50,000; Hidden layers: 3; Neurons/layer: 128; Activation function: tanh; Learning rate: 0.001; Optimizer: Adam; Loss: displacement, velocity, acceleration
LSTM-PINN	Epochs: 10,000; Hidden layers: 3; LSTM layer: 3; Sequence length: 200; Neurons/layer: 128; Activation function: tanh; Learning rate: 0.001; Optimizer: Adam; Loss: displacement, velocity, acceleration

**Table 3:** Numerical results of SDOF structure.

Index	PINN-1		PINN-2		LSTM-PINN	
	Mean Error	MSE	Mean Error	MSE	Mean Error	MSE
Displacement (m)	9.89 × 10^−4^	4.13 × 10^−6^	6.69 × 10^−6^	**0.00 × 10^−0^**	**3.40 × 10^−6^**	**0.00 × 10^−0^**
Velocity (m/s)	3.19 × 10^−3^	3.90 × 10^−5^	2.58 × 10^−4^	6.04 × 10^−7^	**5.97 × 10^−6^**	**1.00 × 10^−9^**
Acceleration (m/s^2^)	3.89 × 10^−3^	6.57 × 10^−5^	2.17 × 10^−5^	3.00 × 10^−9^	**8.53 × 10^−6^**	**2.00 × 10^−9^**

**Table 4:** Character of MDOF structure.

Story	Mass (ton)	Stiffness (kN/m)	Damping (kN·s/m)
10	98	34,310	442.599
9	107	37,430	482.847
8	116	40,550	523.095
7	125	43,670	563.343
6	134	46,790	603.591
5	143	49,910	643.839
4	152	53,020	683.958
3	161	56,140	724.206
2	170	52,260	674.154
1	179	62,470	805.863

**Table 5:** Numerical results of the first floor in the MDOF structure.

Index	PINN-1		PINN-2		LSTM-PINN	
	Mean Error	MSE	Mean Error	MSE	Mean Error	MSE
Displacement (m)	2.16 × 10^−4^	2.33 × 10^−7^	1.51 × 10^−5^	1.08 × 10^−9^	**8.04 × 10^−7^**	**3.57 × 10^−12^**
Velocity (m/s)	1.09 × 10^−3^	4.15 × 10^−6^	3.51 × 10^−4^	3.70 × 10^−7^	**2.89 × 10^−5^**	**5.53 × 10^−9^**
Acceleration (m/s^2^)	6.11 × 10^−2^	2.69 × 10^−2^	1.61 × 10^−2^	2.17 × 10^−3^	**2.22 × 10^−4^**	**1.90 × 10^−7^**

**Table 6:** Numerical results of the 10th floor in the MDOF structure.

Index	PINN-1		PINN-2		LSTM-PINN	
	Mean Error	MSE	Mean Error	MSE	Mean Error	MSE
Displacement (m)	3.64 × 10^−3^	4.83 × 10^−5^	7.76 × 10^−4^	3.84 × 10^−6^	**3.17 × 10^−5^**	**5.63 × 10^−9^**
Velocity (m/s)	4.19 × 10^−2^	5.15 × 10^−3^	1.13 × 10^−2^	5.32 × 10^−4^	**1.13 × 10^−4^**	**5.55 × 10^−8^**
Acceleration (m/s^2^)	4.30 × 10^−1^	8.16 × 10^−1^	8.88 × 10^−2^	4.72 × 10^−2^	**2.66 × 10^−4^**	**2.44 × 10^−7^**

**Figure A1:**
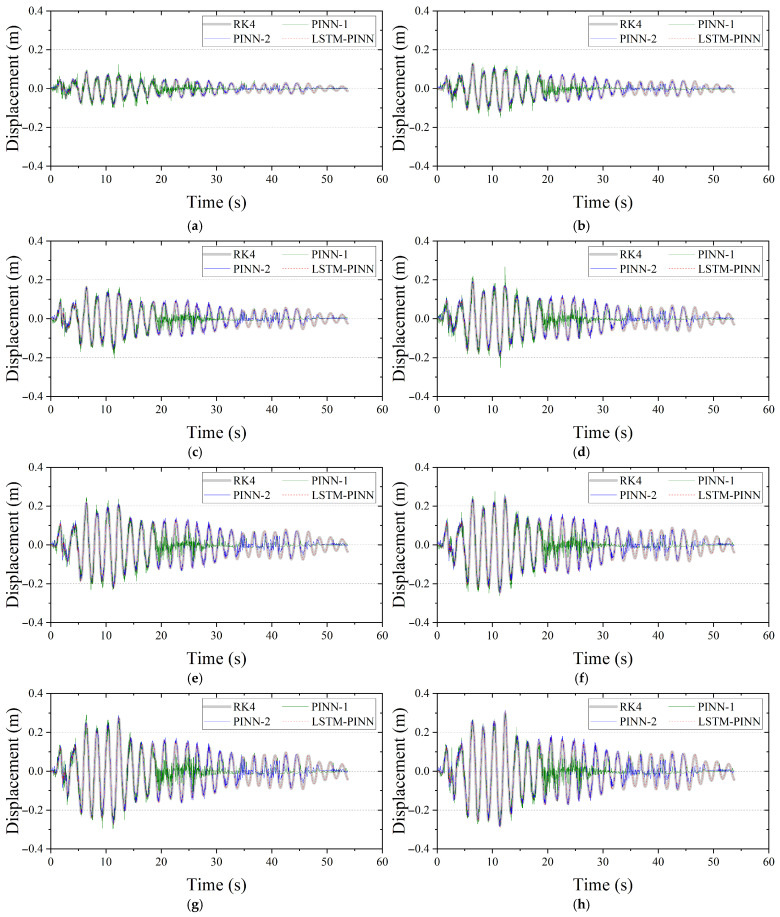
Seismic displacement response of the MDOF structure. (**a**) Second floor; (**b**) third floor; (**c**) fourth floor; (**d**) fifth floor; (**e**) sixth floor; (**f**) seventh floor; (**g**) eighth floor; (**h**) ninth floor.

**Figure A2:**
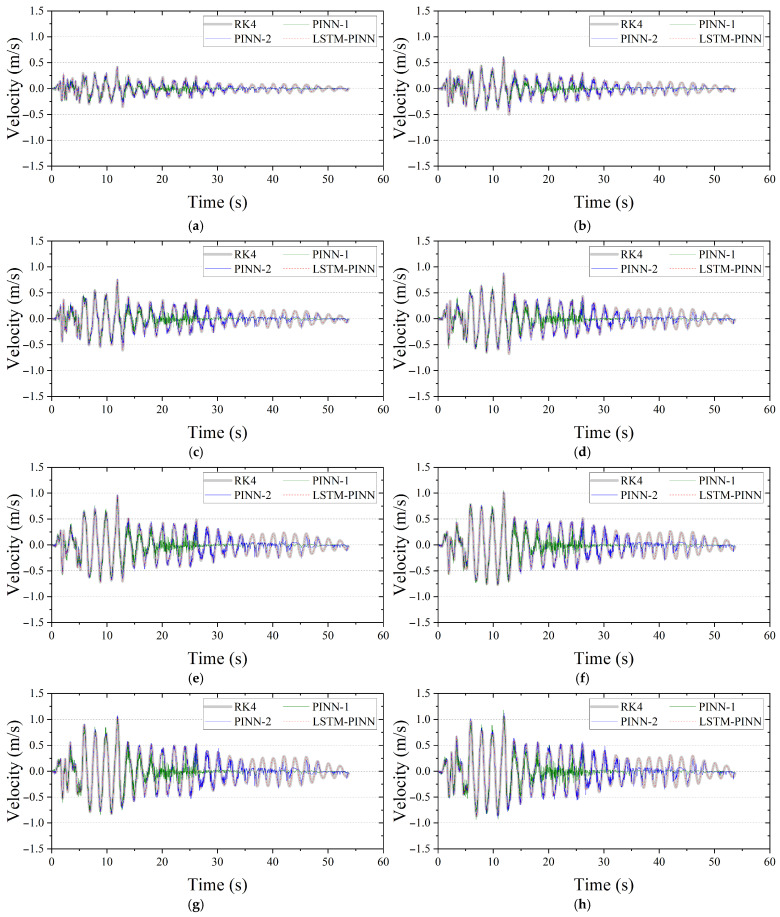
Seismic velocity response of the MDOF structure. (**a**) Second floor; (**b**) third floor; (**c**) fourth floor; (**d**) fifth floor; (**e**) sixth floor; (**f**) seventh floor; (**g**) eighth floor; (**h**) ninth floor.

**Figure A3:**
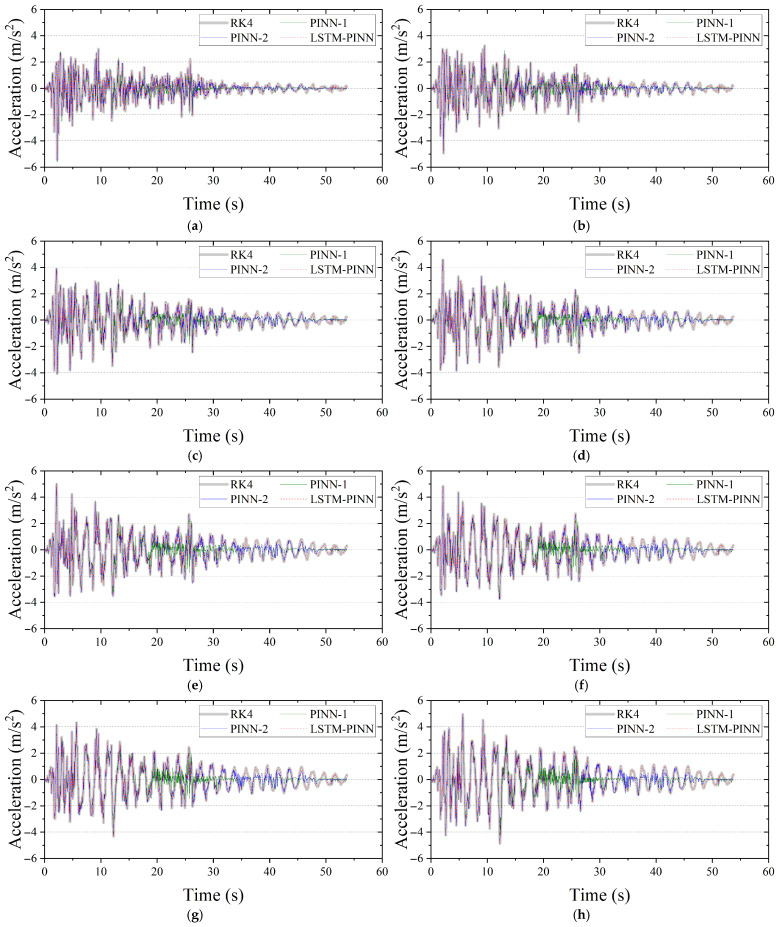
Seismic acceleration response of the MDOF structure. (**a**) Second floor; (**b**) third floor; (**c**) fourth floor; (**d**) fifth floor; (**e**) sixth floor; (**f**) seventh floor; (**g**) eighth floor; (**h**) ninth floor.

**Figure A4:**
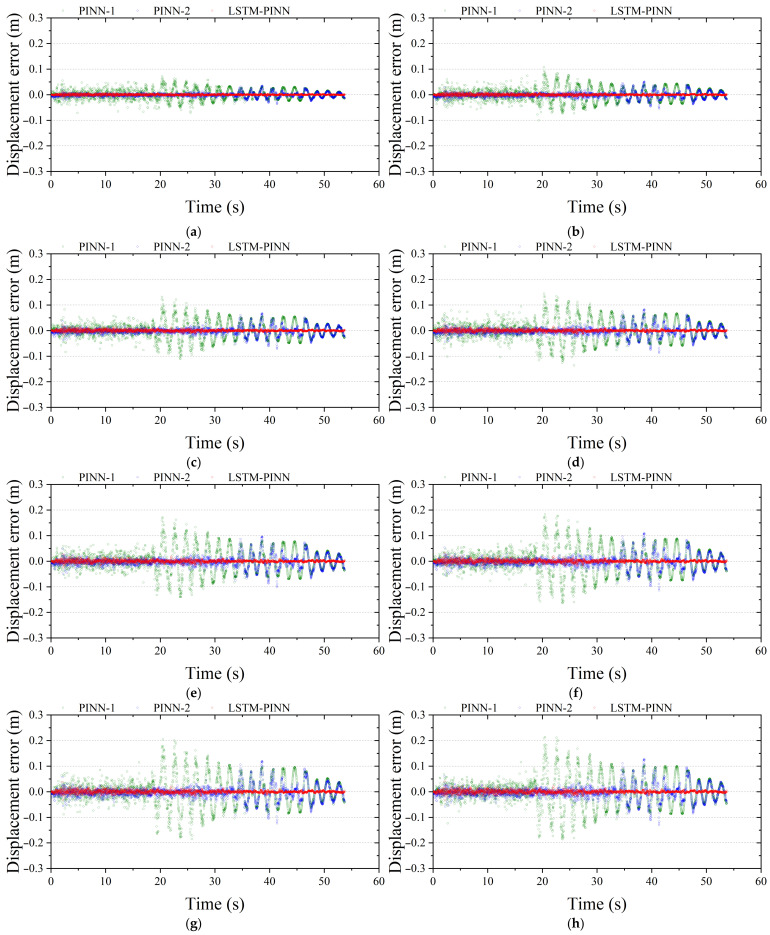
Displacement fitting error analysis of the MDOF structure. (**a**) Second floor; (**b**) third floor; (**c**) fourth floor; (**d**) fifth floor; (**e**) sixth floor; (**f**) seventh floor; (**g**) eighth floor; (**h**) ninth floor.

**Figure A5:**
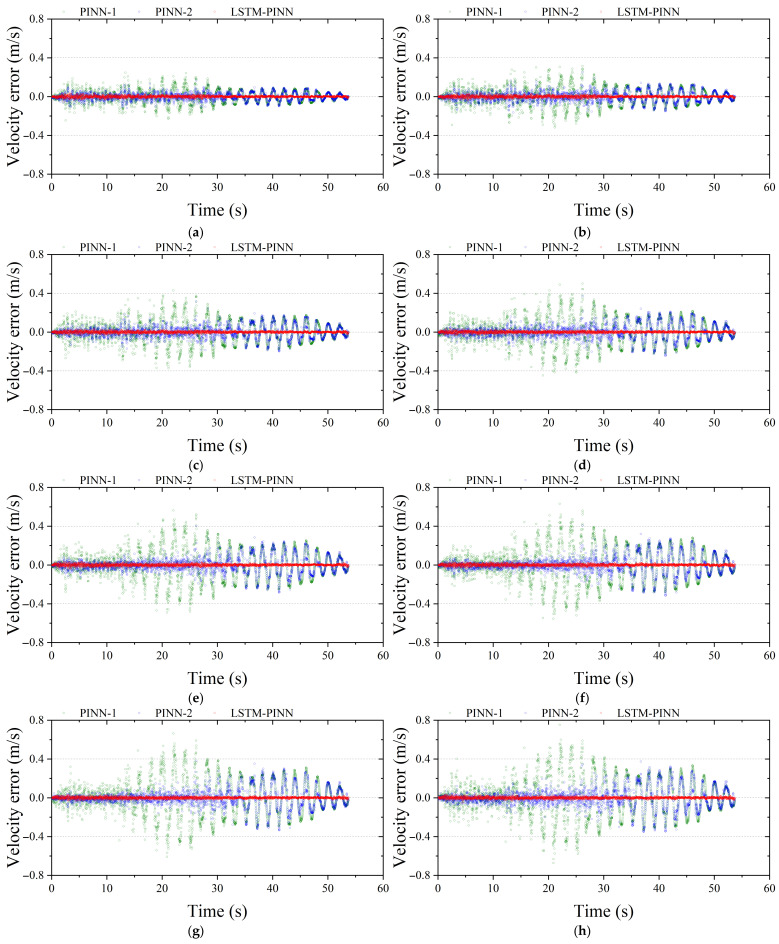
Velocity fitting error analysis of the MDOF structure. (**a**) Second floor; (**b**) third floor; (**c**) fourth floor; (**d**) fifth floor; (**e**) sixth floor; (**f**) seventh floor; (**g**) eighth floor; (**h**) ninth floor.

**Figure A6:**
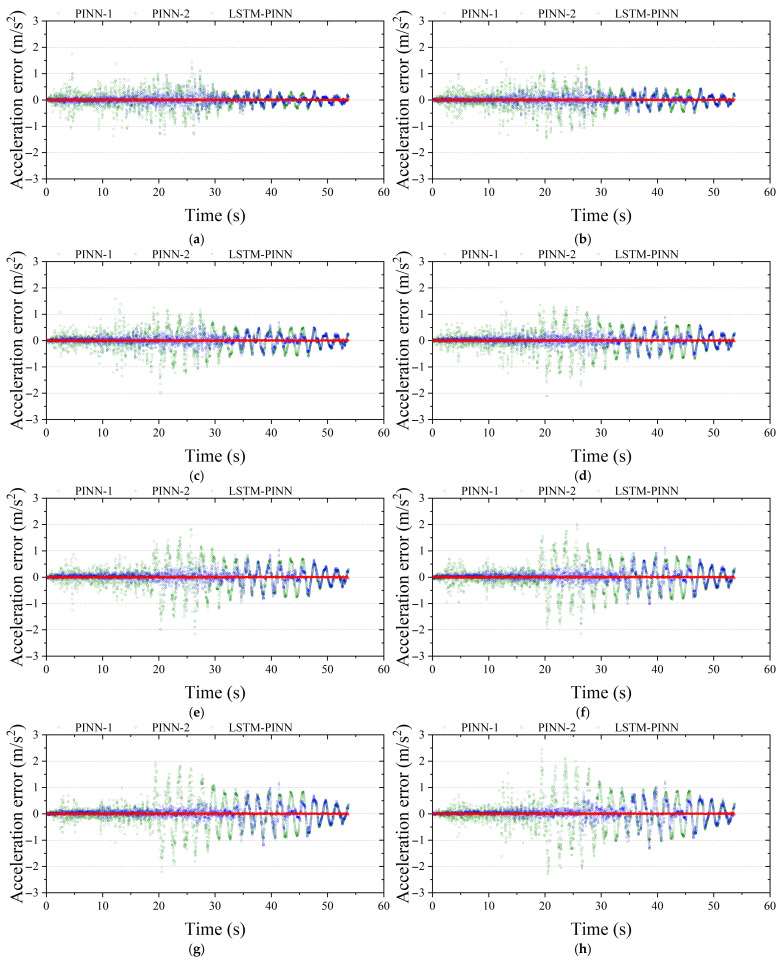
Acceleration fitting error analysis of the MDOF structure. (**a**) Second floor; (**b**) third floor; (**c**) fourth floor; (**d**) fifth floor; (**e**) sixth floor; (**f**) seventh floor; (**g**) eighth floor; (**h**) ninth floor.

## Data Availability

The data is contained within the article. The data from this research can be accessed upon request by contacting the corresponding author.

## Appendix A

Figure A1, Figure A2 and Figure A3 illustrate the displacement, velocity, and acceleration responses for the second to ninth floors of the MDOF structure. Consistent with the results discussed in the main text, the LSTM-PINN model demonstrates the closest agreement with the reference RK4 solution across all response variables.

## Appendix B

Figure A4, Figure A5 and Figure A6 present the prediction errors in displacement, velocity, and acceleration responses for the second to ninth floors of the MDOF structure. In line with the findings reported in the main text, the LSTM-PINN model consistently exhibits the lowest prediction error across all intermediate floors and response variables.
